# Computational and Experimental Validation of B and T-Cell Epitopes of the *In Vivo* Immune Response to a Novel Malarial Antigen

**DOI:** 10.1371/journal.pone.0071610

**Published:** 2013-08-16

**Authors:** Elke S. Bergmann-Leitner, Sidhartha Chaudhury, Nicholas J. Steers, Mark Sabato, Vito Delvecchio, Anders S. Wallqvist, Christian F. Ockenhouse, Evelina Angov

**Affiliations:** 1 Malaria Vaccine Branch, U.S. Military Malaria Vaccine Program, Walter Reed Army Institute of Research, Silver Spring, Maryland, United States of America; 2 U.S. Military HIV Research Program, Division of Retrovirology, Silver Spring, Maryland, United States of America; 3 DoD Biotechnology High Performance Computing Software Applications Institute, Telemedicine and Advanced Technology Research Center, US Army Medical Research and Materiel Command, Fort Detrick, Maryland, United States of America; 4 Vital Probes, Inc., Mayfield, Pennsylvania, United States of America; CSIR-Institute of Microbial Technology, India

## Abstract

Vaccine development efforts will be guided by algorithms that predict immunogenic epitopes. Such prediction methods rely on classification-based algorithms that are trained against curated data sets of known B and T cell epitopes. It is unclear whether this empirical approach can be applied prospectively to predict epitopes associated with protective immunity for novel antigens. We present a comprehensive comparison of *in silico* B and T cell epitope predictions with *in vivo* validation using an previously uncharacterized malaria antigen, CelTOS. CelTOS has no known conserved structural elements with any known proteins, and thus is not represented in any epitope databases used to train prediction algorithms. This analysis represents a blind assessment of this approach in the context of a novel, immunologically relevant antigen. The limited accuracy of the tested algorithms to predict the *in vivo* immune responses emphasizes the need to improve their predictive capabilities for use as tools in vaccine design.

## Introduction

The identification of immune correlates and - moreover- the antigens that induce these protective responses is critical for effective vaccine development. In the post-genomic era, reverse vaccinology approaches, the rational selection of antigens from sequence data, are increasingly used to determine key immunological epitopes [1]. However, immune correlates for many if not most infectious diseases including the knowledge of the antigenic targets of protective immunity are still unknown. Most vaccines currently in the clinic are based on purified, immunodominant antigens or attenuated or inactivated whole pathogens. Such vaccines typically require specialized manufacturing processes and cannot be easily adapted to newly emerging strains. In contrast, a rationally designed, recombinant vaccine based on a single antigen or a small number of antigens, representing several different serotypes, can be produced quickly, cheaply and safely. Advances in *in silico* methods capable of predicting immune epitopes for B cells and T cells will enable the screening of pathogens for immunogenic antigens followed by the determination of epitopes with the highest likelihood of inducing protective immune responses.

B lymphocytes recognize native protein, glycolipids, and polysaccharide antigens based on either a linear epitope or a highly-specific three-dimensional conformational epitope. Continuous linear B cell epitopes can be experimentally mapped using peptide-scanning techniques where overlapping peptides spanning the entire sequence are individually tested for antibody interacting residues. Conformational B cell epitopes, in contrast, are influenced by the physiochemical and structural features of spatially adjacent residues complicating their identification. Unlike B cells, T cells only recognize linear peptide fragments of antigens presented by various MHC molecules on antigen-presenting cells (APC). Current methods for predicting T cell epitopes screen for sequence patterns preferred by the different MHC class I and/or MHC class II alleles with unique peptide binding specificities. Additionally, epitope specificity is conferred by the proteolytic process by which protein antigens are cleaved into peptide fragments within APCs, which depends on the class of APC as well as its activation status (*i.e.*, presence of the proteasome *vs*. immunoproteasome). Algorithms that predict epitope sequences are available for both T cell and B cell epitopes. The *Rankpep* prediction tool considers the binding motifs for MHC class I or II alleles and proteasome cleavage specificities [2]–[4]. Linear B cell epitopes are predicted using computational tools that take into account biochemical properties such as amino acid composition, hydrophobicity, hydrophilicity, surface accessibility, and/or secondary structure. The *Kolaskar-Tongaonka antigenicity* (*KTA*) [5] method is a semi-empirical approach that combines physicochemical properties of an amino acid sequence with its observed frequency in a database of antigenic determinants to predict linear B cell epitopes. *Bepipred* [6] uses a hidden Markov model-based method along with amino acid propensity scales for accessibility, hydrophilicity, flexibility and polarity trained on a dataset of curated B cell epitopes. Lastly, the *ABCpred* prediction tool [7] is an artificial neural network-based B cell epitope prediction server that recognizes that B cell epitopes have varying lengths (5 to 20 residues). *ABCpred* generates datasets of fixed length patterns by eliminating or adding residues at the terminal ends of the peptides.

Discontinuous conformational epitopes, which represent about 90% of all B cell epitopes [8] are much harder to predict requiring knowledge of the antigen’s molecular structure. The *Discotope* computational tool uses antigen protein structure determined by X-ray crystallography or nuclear magnetic resonance (NMR) to predict conformational epitopes using amino acid composition, spatial information, and surface accessibility [9]. However, when an experimentally determined structure of the antigen is unavailable, structure models derived either from homology modeling or from *ab initio* structure prediction can be used.

The aim of the current study was to evaluate computational tools for predicting B and T cell epitopes using the Cell Traversal Protein for Ookinetes and Sporozoites (CelTOS) as the model antigen. CelTOS was identified by genomic and functional analysis of proteins expressed in motile life stages of the malaria parasite *Plasmodium*. It is essential for the parasite’s migration from the mosquito midgut to the salivary gland and in the vertebrae host for migration from the mosquito bite site in the skin to the liver [10]. CelTOS is highly conserved among *Plasmodium* species, but has no known conserved structural elements and no sequence homology to any other known protein. We previously demonstrated that immunization with recombinant CelTOS from *Plasmodium falciparum* (*Pf*CelTOS) induces cross-species protection against murine malaria [11]. Using the heterologous *Plasmodium berghei* murine challenge model of malaria, the protective efficacy of a vaccine can be determined by injecting salivary gland sporozoites subcutaneously to mimic the natural route of infection [12]. Both B and T cell responses contribute to CelTOS-mediated sterile immunity [13] indicating the presence of protective B and T cell epitopes. Since *Pf*CelTOS represents the first malarial antigen with demonstrated cross-species protection, identification of immunogenic epitopes within CelTOS will be crucial to deducing mechanisms of protection. This information will guide the development of improved subunit vaccines for multi-antigen vaccine formulations as well as reveal regions of the antigen possibly involved in immune evasion.

Putative linear B cell epitopes in *Pf*CelTOS were predicted using *KTA*, *Bepipred*, and *ABCpred* methods. While for conformational B-cell epitopes, structural models of CelTOS were first generated using the publicly available protein structure prediction methods, Rosetta [14], I-TASSER [15], and QUARK [16]. The top-ranked structure from each method was then used as input in *Discotope*. We selected *KTA*, *BepiPred*, *ABCpred*, and *Discotope* because they are the most well established, widely used, and heavily cited B cell epitope prediction methods available. However, recently a second generation of epitope prediction methods have emerged that make use of new or improved prediction algorithms and expanded training sets. To evaluate these newer approaches, we carried out additional epitope predictions using the linear epitope prediction methods *BayesB* [17], *CBTope* [18], and *COBEPro* [19], and the conformational epitope prediction methods *ElliPro* [20], *EPSVR* [21], *SEPPA* [22], and *BEPro* [23].

T cell epitopes in *Pf*CelTOS that bind to MHC class I and class II epitopes for C57BL/6 and BALB/c mice were predicted using the *Rankpep* algorithm. *In silico* derived B and T cell epitopes were experimentally verified *in vivo* using *Pf*CelTOS-vaccinated rabbits and mice immune sera. Compilation of these data clearly identified several regions of the antigen harboring B cell epitopes as well as several T cell epitopes that are not genetically restricted. These results highlight immunogenic regions of CelTOS that may be responsible for the observed cross-species protection and are of interest for further immunological characterization. This study marks the first comprehensive, blind assessment of several epitope prediction methods with experimentally derived *in vivo* immune responses for CelTOS.

## Methods

### Ethics Statement

The immunization study was conducted under the approved protocol, 11-MVD-32. “Research was conducted in compliance with the Animal Welfare Act and other federal statutes and regulations relating to animals and experiments involving animals and adheres to principles stated in the *Guide for the Care and Use of Laboratory Animals*, NRC Publication, 1996 edition. All procedures were reviewed and approved by the Institute’s Animal Care and Use Committee (Walter Reed Army Institute of Research), and performed in a facility (Walter Reed Army Institute of Research) accredited by the Association for Assessment and Accreditation of Laboratory Animal Care, International”.

### Protein Structure Modeling

Rosetta [24] was used to carry out *ab initio* structure prediction of *Pf*CelTOS for residues 25–182 (NCBI Reference Sequence: XP_001350569.1), truncating the predicted N-terminal signal sequence. 10^6^ independent structural models were used as an initial set of candidate structures, re-ranked the structures using the DFIRE empirical score function [25], and selected the top 1000 best scoring structures for further refinement using the GB22 score function [26]. Hierarchical clustering using the MMTSB tool set [27] were generated and the cluster centers were selected as representative structure predictions. In addition to using Rosetta, structure predictions were generated using the structure prediction web servers I-TASSER [15] and QUARK, using the same input sequence. The structure predictions from Rosetta, I-TASSER, and QUARK, were used as inputs to predict conformational B cell epitopes using *Discotope* [8].

### B cell Epitope Prediction

The *Pf*CelTOS sequence (residue 25–182) was used as the input for the sequence-based linear B cell epitope predictions using *KTA* [5], *BepiPred* [6], and *ABCpred* [7]. Sequence-based predictions of secondary structure were carried out using the PSI-PRED algorithm [28] and disordered region prediction using the *IUPred* algorithm [29]. To evaluate the newer, second-generation, epitope prediction methods, we used the CelTOS sequence as inputs for *BayesB* [17], *CBTope* [18], and *COBEpro* [19], and the structure prediction from Rosetta as the input for *Ellipro* [20], *EPSVR* [21], *SEPPA* [22], and *BEPro* [23].

### Proteasome and Cathepsin Cleavage

Cathepsin (CAT) D, L, and S were purchased from ENZO life sciences (Farmingdale, NY, USA). THP- 1 cells were purchased from ATCC (Manassas, VA). Proteasomes were isolated from the THP-1 human macrophage cell line, as described in [30]. The concentration of the isolated proteasomes was determined using the (BCA) (Pierce, Rockford, IL). The composition of the isolated proteasomes was determined by two-dimensional isoelectrophoresis/SDS-PAGE (2-D IEF) followed by Western Blot detection of either the constitutive proteasome subunits or the immunoproteasome subunits (ENZOLifeSciences) [30], [31].

### Enzymatic Degradation of CelTOS

Based on previously described methods [30], [31] 10 µg of CelTOS were incubated with purified proteasomes (1 µg) or with each of the respective CAT (0.5 µg) for 16 hrs at 37°C unless otherwise stated. To ensure the specificity of proteasomes, epoxomicin was used as an inhibitor (EnzoLifeSciences, Farmingdale, NY). The inhibitor was pre-incubated with the proteasomes before the addition of the antigen. The reactions were stopped by freezing the samples at −80°C. The proteasomal and CAT degradation products were analyzed on an LCMS-IT-TOF mass-spectrometry (Shimadzu, Columbia, MD). A separate aliquot of the degradation products was analyzed by SDS-PAGE and stained with GelCode Reagent Blue Stain (Pierce).

### Separation and Analysis of Peptides

The CAT and proteasomal degradation products of *Pf*CelTOS were analyzed by LCMS-IT-TOF mass spectrometry by MS and tandem MS/MS as previously described [30], [32]. Each sample was analyzed in duplicate. Peptides were identified using the Mascot Software (Matrix Science, London, UK) with the MS/MS ion search. The peptide MS tolerance was set to 0.2 Da and the MS/MS tolerance was set to 0.1 Da using the monoisotopic peaks. Searches were conducted using the known sequence of *Pf*CelTOS and the Swiss-Prot database.

### Subtractive MALDI-TOF Analysis of Immune Sera

A peptide library of forty-three *Pf*CelTOS synthetic 15-mer peptides overlapping by eleven amino acids (Mimotopes Pty Ltd, Clayton, Australia, ProImmune (ThinkPeptides®), Bradenton, FL; purity >95% based on HPLC) was used for subtractive-MALDI analysis. Pre-immune and pre-challenge (immune) sera from BALB/c-J mice (Jackson Laboratories, Bar Harbor ME) were incubated with each of the individual peptides (1 µg) at both 37°C for 1 hr as well as 4°C for 4 hrs at dilutions of 1∶1,000 and 1∶10,000 in 10 µL 50 mM Tris HCl (pH 7.8) containing 150 mM NaCl. The matrix surface was created by applying 1 µL of α-cyano-4-hydroxycinnamic acid in a 70∶30 mixture of acetonitrile and 1% trifluoroacetic acid to a 384-well plate (KRATOS Analytical, Chestnut Ridge NY) and heat-dried at 37°C for 5 min. An aliquot of each reaction mixture (peptide only as baseline, peptide incubated with pre-immune sera as negative control, and peptide incubated with immune sera) was added to the matrix bed and allowed to dry at 37°C for 10 min. Excess salts were removed by adding 5 µL of ultra pure water (EMD Millipore Chemicals, Rockland MA) and pipetted off after 30 seconds. Finally an additional 1 µL of α-cyano-4-hydroxycinnamic acid was overlaid on the sample as described and allowed to heat dry at 37°C for 10 min. Subtractive-MALDI analysis was completed with an Axima CFR-Plus time-of-flight mass spectrometer (Shimadzu Biotech, Columbia MD) using a 337 nm nitrogen laser operating in positive ion mode with an accelerating voltage of +20 kV and an extraction delay of 500 ns. Spectra were generated using a laser power of 74–77 averaging 75–150 shots per profile and compiling the average for improved data quality. Peak areas were measured using the KRATOS software package to determine which peptides were positive for binding with the immune antibodies.

### Immunizations

Recombinant *Pf*CelTOS with endotoxin levels below detection was manufactured as described earlier ([11]. Mice used for immunizations were 6- to 8-week-old female BALB/c-J, C57BL/6 (Jackson Laboratories, Bar Harbor ME) or ICR from Charles River Laboratories (Wilmington MA). Mice were immunized using a regimen previously shown to induce protective immunity (10 µg/dose of recombinant *Pf*CelTOS emulsified in Montanide ISA 720 (Seppic Inc., Fairfield NJ) [33]. New Zealand white rabbits were immunized three times intramuscularly, at three-week intervals with 50 µg *Pf*CelTOS/Montanide ISA-720 (Spring Valley Laboratories, Inc., Sykesville, MD).

### ELISA

Immulon 4 ELISA plates (Thermo Scientific, Waltham, MA) were coated with peptides (same as were used for the MALDI-TOF analysis; 10 µg/mL) diluted in PBS overnight at 4°C. After washing with PBS/0.1% Tween 20, the plates were blocked with 1% bovine serum albumin/PBS/Tween 20. Antisera from eight individual rabbits or pooled sera from BALB/C immunized three times with recombinant *Pf*CelTOS antigen adjuvanted in Montanide ISA-720 were diluted (1∶200) in PBS/0.1% BSA and incubated in duplicate in peptide-coated ELISA plates for 1 hr at RT. After washing, plates were incubated with HRP-labeled secondary antibodies against rabbit or mouse IgG (KPL Gaithersburg, MD) for 1 hr at RT. ABTS substrate (Thermo Scientific, 50 µL/well) was added to each well for 1 hr at RT. Optical density was read on an ELISA plate reader at 405 nm. Data are represented as the (mean OD of immune sera) - (mean OD of normal mouse sera or pre-immune rabbit sera).

### ELISpot

ELISpot assays (R&D Systems, Minneapolis, MN) were performed as described earlier [11].

### Classification of B cell Epitopes of CelTOS from Experimental Data

Classification of a residue as epitope or non-epitope based on the peptide scanning data is important for properly assessing the computational B cell epitope prediction algorithms. In peptide scanning, overlapping peptides are used to probe serum antibody binding, and each residue is present in several overlapping peptides, each with different observed binding affinities (measured as optical density, or OD) to serum antibodies. For simplicity, a given residue’s score from the peptide-scan of mouse or rabbit antisera was defined as the maximum OD value observed for the set of overlapping peptides it was present in. OD value thresholds of 0.2 and 0.4 were used for mouse and rabbit anti-sera, respectively, above which a residue was classified as an epitope residue. These thresholds reflect OD measurements for CelTOS peptides representing the N-terminal signal sequence which was absent in the recombinant *Pf*CelTOS construct used for immunization and thus considered to be not antigen-specific.

### Assessment of B cell Epitope Mapping Predictions

Quantitatively comparing predicted and experimental epitope mapping results is critical for the proper assessment of their accuracy and significance. An adapted approach [6] was applied to calculate both the accuracy and the statistical significance of the mapping data. B cell epitope prediction methods output scores for each antigen residue that reflects the propensity for that residue to be an epitope residue. For each method, the respective published score cutoff recommendation was used to classify each residue as an epitope residue (*ABCpred* score ≥0.51; *Bepipred* score ≥0.35; *Discotope* score ≥ −7.7; *KTApred* score ≥1.0). For *ABCpred*, where contiguous peptide segments are scored, we defined the residue score as the maximum peptide score for which that residue was a part. The mouse epitope mapping data was used to define the ‘true’ B cell epitopes for CelTOS. All other methods: *ABCpred*, *Bepipred*, *Discotope*, *KTApred*, and the rabbit peptide-scan residue scores (see above), were assessed as predictive methods.

The accuracy of each B cell epitope prediction method was calculated as the percentage of correctly classified (epitope vs. non-epitope) residues in the CelTOS protein sequence. While this measure is simple and intuitive, it cannot capture the degree of specificity or sensitivity of the predictive method. A better metric for B cell epitope prediction accuracy is the area under the curve generated by the receiver operator characteristic (ROC), known as A_ROC_ (reviewed in [34]). The ROC curve is a function of both sensitivity and specificity, is invariant with respect to the proportion of positives and negatives in the data set, and does not require a cutoff score to assess predictive accuracy. A perfect prediction results in an A_ROC_ value of 1, while a completely random prediction results in an A_ROC_ value of 0.5.

The reliability of the mapping results in terms of the standard error (SE) of the A_ROC_ value and a *p*-value of the A_ROC_, describing the probability of arriving at that A_ROC_ value by chance, was estimated using a bootstrapping approach as described in Larsen *et al*. [6]. For a given epitope prediction set, a large number of ‘pseudo-replica’ data sets were generated consisting of random sampling and random permutations of the original data set, for SE and *p*-value estimation, respectively. A_ROC_ values were calculated for each pseudo-replica data set. The SE of these A_ROC_ values was used to estimate the true SE of the epitope prediction set. The *p*-value of the observed A_ROC_ value for a given epitope prediction set was estimated as the probability with which the observed A_ROC_ value occurred in the pseudo-replica sets.

## Results

### *In vivo* Epitope Mapping of the Humoral Immune Response in Rabbits and Mice

**Figure 1:**
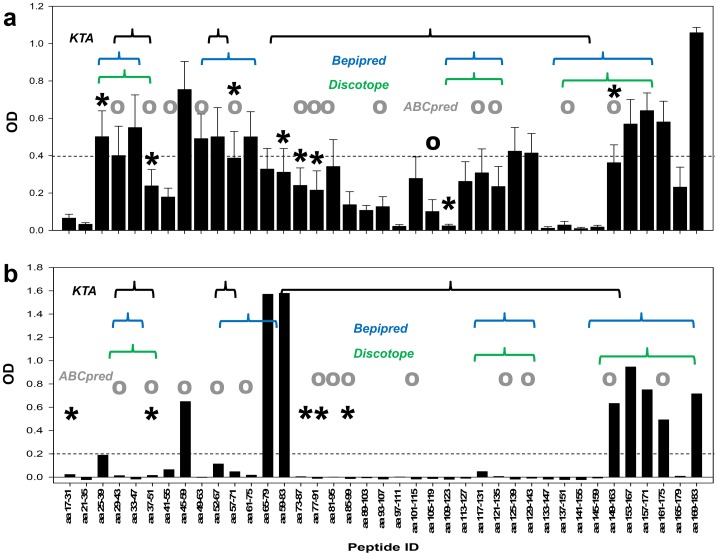
Comparison of specificity of *in vivo* immune responses with *in silico* predictions using various algorithms. Individual rabbit (Panel A, n = 8 animals) or mouse (Panel B, n = 10 animals) immune sera were analyzed by ELISA and reported as OD values (bar graph). X-axis indicates the peptide ID and span; Y-axis indicates the OD value of the ELISA after subtracting the background measured for pre-immune sera. Horizontal line indicates threshold OD as described in Materials and Methods. Brackets indicate the segments of the protein predicted to be immunogenic by the *KTApred* (black), the *Bepipred* (blue) or the *Discotope* (green) algorithms. Open circles (grey) above individual bars identify peptides predicted by the *ABCpred* prediction tool. Asterisks above individual bars indicate positive responses detected by MALDI-TOF MS analysis. The MALDI-TOF data are the mean of three independent experiments using pooled immune sera. Responses from pooled pre-immunes were subtracted.

*Pf*CelTOS immune rabbit sera were tested for their reactivity to overlapping 15-mer peptides by ELISA and MALDI-TOF (Figure 1A). The peptides overlap by eleven amino acids and span the entire *Pf*CelTOS sequence. Five distinct regions were identified as being immunoreactive: AA 25–55, AA 45–100, AA 105–115, AA 113–143, and AA 149–183. Peptides AA 25–39, AA 37–51, AA 61–75, AA73–91, AA 85–99, AA 117–131 and AA 161–175 were recognized by the rabbit sera in agreement with the specificity obtained in the ELISA. Characterization of immune mouse sera by ELISA and MALDI-TOF (Figure 1B) revealed that the antibody responses were skewed toward the N-terminus (residues 25–39), with two distinct regions near the N-terminus (residues 45–59 and residues 65–83), and an extended segment along the C-terminus (residues 149–183). The MALDI-TOF MS data did not significantly overlap with results from ELISAs. Overall, epitope mapping of the rabbit sera was in good agreement with the mouse ELISA data. The N-terminal-most epitope and the long C-terminal epitope region were identified in both sera. Mouse sera identified an epitope (peptides AA65–83) that was not recognized by rabbit sera, while rabbit sera identified several epitopes (peptides AA125–139 and AA129–143) that were not seen by mouse sera. Hereafter, the N-terminal and adjacent to-N-terminal epitopes found from the rabbit or mouse serological data will be referred to as epitope I (residue 25–83), which consists of three epitope sub-regions, termed Ia (residues 25–44), Ib (residues 45–59), and Ic (residues 61–83). The epitope corresponding to residues 125–143 in the rabbit data will be referred to as epitope II. Finally, the long C-terminal epitope (residues 149–183) will be referred to as epitope III.

### Computational Predictions of Linear and Conformational B cell Epitopes

**Figure 2:**
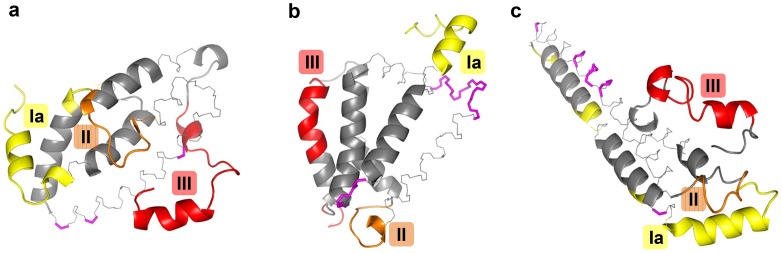
Top-ranked structure predictions of the CelTOS protein using Rosetta (A), i-TASSER (B), and QUARK (C) as backbone traces. Predicted conformational epitopes by *Discotope* are shown as colors for epitopes I (yellow), II (orange), and III (red). Regions predicted to be epitopes by *Discotope* but not found to be antigenic in peptide scans are shown in magenta.

The protein sequence of *Pf*CelTOS was used to predict linear B cell epitopes using the sequence-based *KTA*, *Bepipred*, and *ABCpred* algorithms. Prediction of conformational B cell requires structural models of *Pf*CelTOS. Towards that end we used the computational structure prediction programs Rosetta, I-TASSER, and QUARK to model the tertiary structure of *Pf*CelTOS from its protein sequence (Figure 2). Rosetta *ab initio* modeling generated a diverse set of predictions, but failed to converge towards a single structure. Structure models by I-TASSER similarly showed high diversity, with few similarities between structures. QUARK, by contrast, showed significant convergence towards an α-helical, coiled-coil hairpin-like conformation that places the N- and C-termini near each other. All structure predictions were high in α-helical character, consistent with previous circular dichroism data [13], and sequence analysis of α-helical regions displayed distinct amphipathic character suggesting that appropriate helical packing is critical to the tertiary structure of CelTOS. Top-ranked structures from each method were used to predict conformational B cell epitopes using the structure-based *Discotope* algorithm.

### Quantitative Comparison between B cell Epitope Predictions and in vivo Epitope Mapping

**Figure 3:**
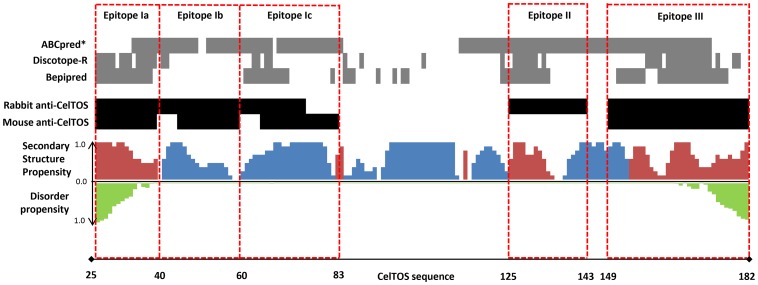
Consolidation of structural properties from *in silico* predictions and *in vivo* immune responses. Computational epitope predictions (gray) are shown for *ABCpred*, *Discotope*, and *Bepipred*. Experimental epitope mapping using antibody peptide scanning (black) from both rabbit and mouse anti-*Pf*CelTOS serum antibodies are significant above an OD-cutoff of the mean background responses plus three standard deviations from the mean. Computational secondary structure propensities for α-helix (blue), coiled (red) regions and disordered propensity (green) reported in a relative scale −1 to 1. All computational epitope definitions are based on classifications using default score cutoff values for *Discotope* and *Bepipred*. Score cutoff for *ABCpred* was optimized to maximize accuracy. Only results from computational methods with statistically significant predictions (*p*<0.001) are shown.

The findings from the *in silico* B-cell predictions and the *in vivo* results obtained in rabbits and mice are mapped over the CelTOS protein sequence in Figure 3 and summarized in Table 1 using two measures of accuracy (accuracy and A_ROC_) and statistical significance (*p*-value). Accuracy reflects the percentage of correctly classified epitope residues. A_ROC_ is a related measurement of accuracy that accounts for sensitivity and specificity and ranges from 0.5 to 1.0 for completely random and completely accurate predictions, respectively. Finally, the *p*-value estimates the probability that such a result would have been observed by chance (see Methods for details).

**Table 1:** B-cell epitope mapping accuracy to mouse anti-*Pf*CelTOS serum antibodies.

Method	Type	Input	Accuracy (%)^a^	A_roc_(±SE)^b^	*p*-value^c^
***Computational epitope prediction***					
*KTApred*	linear epitope prediction	sequence	32.8	0.26±0.04	1.0
*ABCpred*	linear epitope prediction	sequence	44.9	0.64±0.05	<0.001
*Bepipred*	linear epitope prediction	sequence	61.4	0.68±0.05	<0.001
*Discotope-T*	conformational epitope prediction	homology model; I-TASSER	53.8	0.54±0.05	0.498
*Discotope-Q*	conformational epitope prediction	*ab initio* model; QUARK	54.0	0.61±0.05	0.011
*Discotope-R*	conformational epitope prediction	*ab initio* model; Rosetta	58.9	0.67±0.04	<0.001
***Experimental epitope mapping***					
rabbit MALDI-TOF	mass spectrometry peptide scan	rabbit anti-*Pf*CelTOS serum	63.9	0.64±0.04	<0.001
mouse MALDI-TOF	mass spectrometry peptide scan	mouse anti-*Pf*CelTOS serum	51.9	0.53±0.04	0.197
Rabbit pepscan	ELISA peptide scan	rabbit anti-*Pf*CelTOS serum	76.6	0.93±0.02	<0.001
Mouse pepscan	ELISA peptide scan	mouse anti-*Pf*CelTOS serum	(*Comparator used to define accuracy*)		

a Accuracy measures the percentage of correct epitope classification across all residues.

b A_roc_ is the area under the curve constructed by the Receiver Operational Characteristics (ROC), which is the function of the sensitivity and specificity of the epitope mapping score. A_roc_ = 1 indicates perfect prediction of epitopes, A_roc_ = 0.5 indicates completely random predictions.

c p-value is the probability that the observed A_roc_ value was obtained by chance. In the null positive and null negative methods, all and no residues, respectively, were classified as epitopes.

A_roc_ standard errors (SE) were estimated using bootstrapping. *p*-values were calculated using permutation tests [6].

Murine peptide scan data was used to classify B cell epitopes of CelTOS since correlates of immunity can be identified using this model (Table 1). The rabbit peptide scan data represents an alternate species model of the murine immune response in the same way that many animal models are used to predict human immune responses. As such, the accuracy of the rabbit serological data, as an experimental model for epitope mapping, could be considered a gold-standard with which to compare the computational epitope predictions. Overall, the rabbit epitope mapping showed 77% agreement and an A_ROC_ of 0.93 (*p*<0.001) with the mouse epitope classification, indicating a high degree of agreement with mouse serological data, as expected.

Linear B cell epitopes were best predicted by *Bepipred* yielding 61% agreement with mouse serological data with an A_ROC_ of 0.68 and a significance of *p*<0.001. It correctly identified a large segment of the N-terminal epitope Ia and the C-terminal epitope III as well as a significant portion of the epitope Ic. *Bepipred* also identified a significant portion of epitope II, which was not recognized by the mouse serum, but reacted with the rabbit serum. *ABCpred* showed high predictive value with an A_ROC_ of 0.64 (*p*<0.001) but with a poor classification accuracy (45% agreement), suggesting that the standard cutoff value used by *ABCpred* to define epitope residues is poorly suited for *Pf*CelTOS, and that relative, rather than absolute scores should be used. *ABCpred* predicted most of epitope I, and significant portions of epitopes II and III. Finally, *KTA* showed no significant predictive value (*p = *1.0).

In conformational epitope prediction using *Discotope*, there was some agreement between the predicted models despite diversity in the overall structures. Specifically, the N-terminal epitope Ia, a significant portion of epitope II, and the C-terminal epitope III were predicted as epitopes by all structure models. Interestingly, although there was significant divergence in the overall protein fold by the structure predictions, the local structure of the predicted B cell epitopes was remarkably consistent across predictions (Figure 2). Epitope prediction using *Discotope* with the Rosetta *ab initio* structural model performed comparably to *Bepipred*, at 59% accuracy with an A_ROC_ 0.67 and a significance of *p*<0.001. Like *Bepipred*, it identified the N-terminal and C-terminal epitope regions (epitopes 1a and III, respectively), as well as a significant portion of epitope II, and portions of epitope 1c. By contrast, *Discotope* using the QUARK *ab initio* structural model showed only modest predictive value, with an A_ROC_ of 0.54 and *p = *0.011, while *Discotope* using the iTASSER homology model showed no significant predictive value (*p = *0.5). The limited accuracy of the predictive methods was not dependent on the animal model as comparing the results with the immune response in rabbits did not result in significantly higher values: 59%, 65% and 59% for *ABCpred*, *Bepipred* and *Discotope*, respectively.

We also carried out B cell epitope predictions using a selection of second-generation prediction methods (Table 2). Among linear epitope prediction methods, *CBTope* performed the best with 58% and an A_ROC_ of 0.58 (*p = *0.03), respectively, while in conformational epitope prediction, *Ellipro* achieved the highest accuracy with 66% while *BEPro* achieved the highest A_ROC_ of 0.73 (*p<*0.001). Overall the results of these newer methods did not show significant improvement over the older *Bepipred*, *ABCpred*, and *Discotope* methods in predicting B cell epitopes in CelTOS.

**Table 2:** B-cell epitope prediction using second generation methods.

Method	Type	Input	Accuracy (%)^a^	A_roc_(±SE)^b^	*p*-value^c^
***Computational epitope prediction***					
*BayesB*	linear epitope prediction	sequence	46.9	0.45±0.02	1
*CBTope*	linear epitope prediction	sequence	58.3	0.58±0.03	0.031
*COBEPro*	linear epitope prediction	sequence	52.5	0.58±0.04	0.044
*EPSVR*	conformational epitope prediction	*ab initio* model; Rosetta	58.3	0.65±0.04	<0.001
*SEPPA*	conformational epitope prediction	*ab initio* model; Rosetta	57.6	0.69±0.04	<0.001
*BEPro*	conformational epitope prediction	*ab initio* model; Rosetta	56.9	0.73±0.04	<0.001
*Ellipro*	conformational epitope prediction	*ab initio* model; Rosetta	64.8	0.68±0.04	<0.001

a Accuracy measures the percentage of correct epitope classification across all residues.

b A_roc_ is the area under the curve constructed by the Receiver Operational Characteristics (ROC), which is the function of the sensitivity and specificity of the epitope mapping score. A_roc_ = 1 indicates perfect prediction of epitopes, A_roc_ = 0.5 indicates completely random predictions.

c p-value is the probability that the observed A_roc_ value was obtained by chance. In the null positive and null negative methods, all and no residues, respectively, were classified as epitopes.

A_roc_ standard errors (SE) were estimated using bootstrapping. *p*-values were calculated using permutation tests [6].

Finally, to ensure that observed agreements between the epitope predictions and the experimental results are significant, two null model predictions were analyzed in which all (positive) and no (negative) residues were classified as epitope residues. While the null models correctly classified 53% and 48% of the residues in the mouse data, they both had A_ROC_ values of 0.50 and *p*-values of 1.0, confirming that their predictive value was no better than chance and underscoring the importance of using more sophisticated metrics such as A_ROC_ and *p*-value rather than simple classification accuracy to measure predictive power.

### Mapping the Cellular Immune Response in Mice

**Figure 4:**
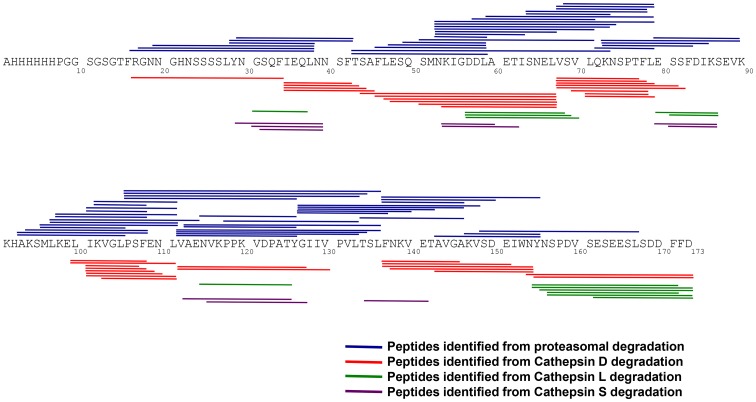
Proteasomal and Cathepsin cleavage maps of CelTOS. The proteasomal and Cathepsin D, L and S peptide fragments were separated by UFLC, analyzed on an LCMS-IT-TOF mass spectrometer and then identified using the MASCOT data base. The peptides derived from the proteasomal degradation of CelTOS (blue lines) are denoted above the sequence, and Cathepsin D (red lines), Cathepsin L (green lines) and Cathepsin S (purple lines) are denoted below the sequence, represent the identified peptides. The data shown is representative of two separate experiments.

Experimental proteasomal cleavage with human proteasomes and cathepsins [30]–[32] was used to identify potential T cell epitopes on *Pf*CelTOS. Studies have demonstrated differential epitope generation depending on whether antigens were cleaved by either constitutive proteasomes or immunoproteasomes. The *Pf*CelTOS protein was subjected to proteasomal cleavage to identify peptides that could potentially bind to MHC molecules and thus function as epitopes. The resulting peptides were identified based on the mass/charge ratio and sequence verification of the fragmentation patterns. Peptide-maps of the MHC class I and MHC class II precursor epitopes were constructed (Figure 4). Seventy-eight peptides were generated from the proteasomal degradation of *Pf*CelTOS, with 87% sequence coverage predominantly at the N-terminus and the central region of the molecule, while 35 peptides were generated from the Cathepsin D degradation of *Pf*CelTOS with 78% sequence coverage, 13 peptides were generated from the Cathepsin L degradation of *Pf*CelTOS with 39% sequence coverage, and 10 peptides were generated from the Cathepsin S degradation of *Pf*CelTOS with 31% sequence coverage. Unlike proteasomal cleavage, Cathepsin cleavage appeared more focused and localized to specific segments of the molecule.

**Figure 5:**
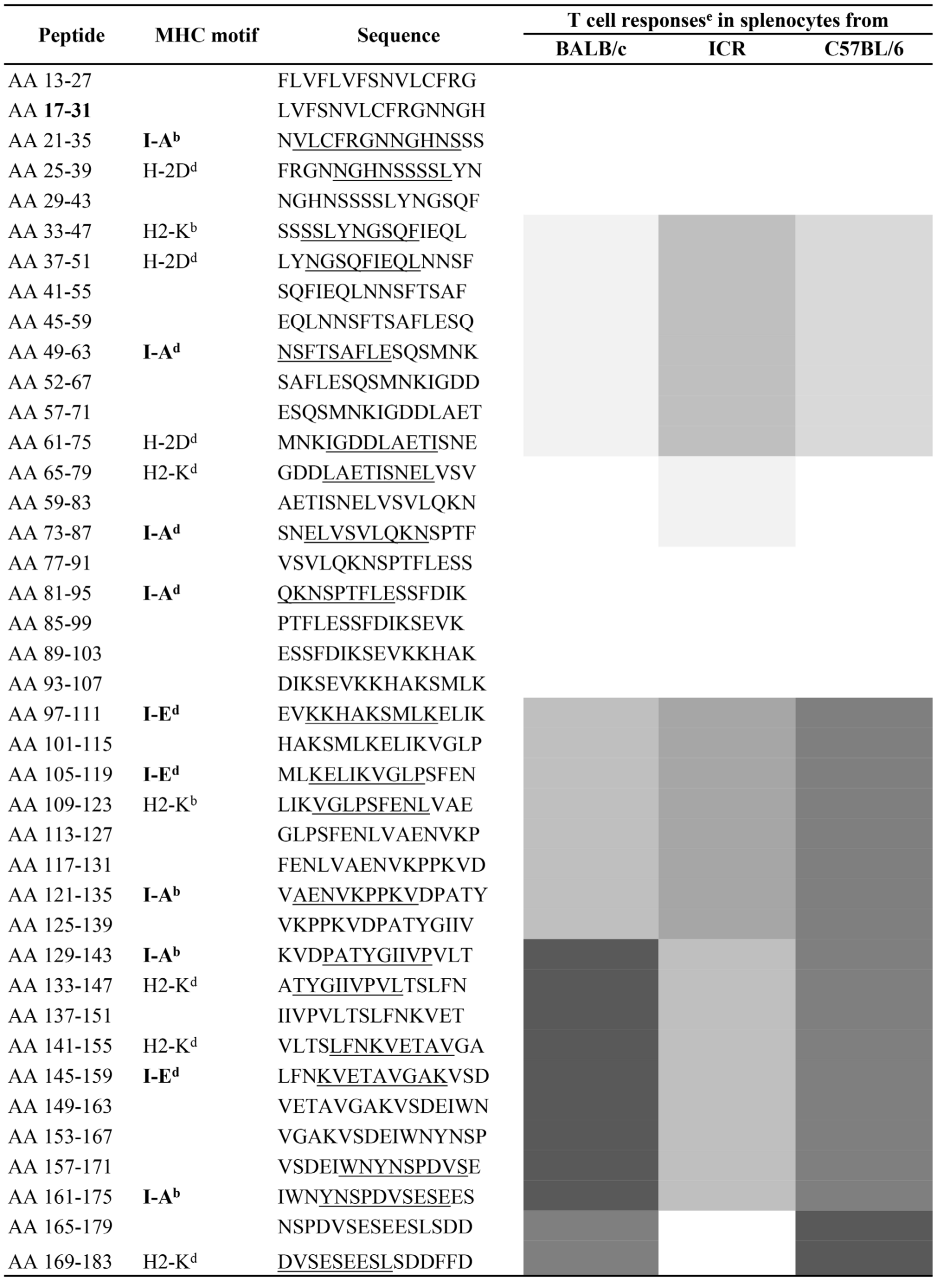
Fine specificity of PfCelTOS-specific T cells. Reactivity was determined by ELISpot analysis measuring *Pf*CelTOS-specific IFN-γ responses. Mouse splenocytes from three strains (inbred BALB/c and C57BL/6 and outbred ICR) were tested against a panel of 43 overlapping peptides (AA = amino acid position within the protein). Putative binding to indicated MHC class I and class II (in bold) alleles was determined by Rankpep analysis. Underlined amino acids designate predicted binding motif for indicated MHC allele. Shading and intensity of shading indicates the magnitude of the T cell response after *ex vivo* stimulation with the peptides.

The *Rankpep* algorithm was applied to predict peptides that bind to MHC class I and class II molecules for C57BL/6 and BALB/c mice in order to predict T cell epitopes (Figure 5). Experimentally-derived responses were deduced using overlapping peptides spanning the entire *Pf*CelTOS protein and *ex vivo* stimulation of cytokine production in splenocytes from BALB/c, C57BL/6 and ICR mice immunized with the recombinant *Pf*CelTOS protein, which induces protective immunity [35]. Although predicted MHC class I and II-restricted epitopes evenly distribute throughout the protein sequence, the C-terminal portion of the protein appeared to be the most immunogenic for all three mouse strains.

## Discussion

The identification of target antigens for the design of a prophylactic vaccine is complicated when immune correlates of protection are unknown. In recent years, reverse vaccinology. *i.e.,* rational vaccine design approaches based on sequence data has been increasingly applied. Functionally and structurally crucial elements of the molecule are used to guide the design of peptide subunit vaccines. For instance, structural elements such as the GPI-anchor of protozoan antigens can interfere with eukaryotic expression in DNA vaccination [36]. The attachment of glycans at putative N-glycosylation motifs in bacterial or protozoan antigens expressed by eukaryotic cells can lead to aberrant glycosylations that can result in either structural changes of crucial epitopes or enhanced phagocytosis [37], [38]. Furthermore, insights into the central role or function of the target antigen in the pathogen will guide vaccine design strategies toward humoral or cellular immunity and may reveal the immune effectors of protection.

The present study sought to identify immunogenic lymphocyte epitopes from a parasite-derived antigen by using a variety of prediction tools and compare these results to *in vivo* immune responses. Development of algorithms that can predict with a high degree of accuracy immunodominant epitopes on protein antigens will greatly enhance vaccine design and development. Currently computational methods are available for predicting B cell and T cell epitopes, but few, if any, have been applied prospectively in a blind assessment. Here, we applied several B cell epitope prediction tools to the novel malaria antigen, CelTOS, whose structure is unknown and whose sequence has no known homology to any functional motifs in the protein database (PDB). The results obtained by the *in silico* methods were compared to the immunogenicity data obtained in two relevant preclinical models [11], [35].

Although the *in silico* methods employed represent the current state of the art for epitope prediction, results from the algorithms used to predict linear B cell epitopes (*Kolaskar* and *Bepipred*) did not significantly overlap with each other (Table 1). The *ABCpred* artificial neuronal network predicted 14 epitopes which matched epitopes identified experimentally in immune rabbit serum. However, although in good agreement, not all peptides recognized by rabbit antibodies were predicted by *ABCpred*. In contrast, only 5 of the 14 epitopes predicted by both immune antibodies and *ABCpred* were detected by MALDI-TOF mass spectra analysis. Here, peptide epitopes were identified by comparing peptide antibody-control (pre-immune) interactions with the peptide immune-antibody interactions. Disappearance of peptide-ion signals in the mass spectra from the antibody reaction mixtures was interpreted as a result of antibody-binding to the epitope sequences on linear peptides. The overall lower numbers of epitopes identified by this method may be partly due to the linear nature of the peptides thus ignoring conformational epitopes.

Discontinuous B cell epitopes predicted by *Discotope* overlapped with the linear epitopes predicted by the *Bepipred* algorithm. Although B cell epitope predictions for linear as well as discontinuous epitopes revealed good matches with the humoral immune responses induced in rabbits, the same tools had an accuracy of only ∼50% when comparing the predictive models with the experimental results in mice. Immune responses targeting to the C-terminus of CelTOS and the relatively accurate prediction of B cell epitopes within this same region are biologically relevant since genetic diversity and single nucleotide polymorphisms (SNPs) in the *celtos* gene of *Plasmodium falciparum* isolates from Central- and South America, Africa and Asia are localized to the same region [39]. These SNPs are concentrated into two distinct regions of the *celtos* gene. One cluster coincides with the C-terminal B-cell dominant epitope predicted by *Discotope* (Figure 1,3) suggesting that this may be a region of immune escape by the parasite and further highlighting its’ important role in protection.

In addition to the widely used *ABCpred*, *Bepipred*, and *Discotope* algorithms, we tested several second generation B cell epitope prediction methods as well. Disappointingly, the results of the new methods did not show significant improvements over the previous methods. This may suggest that these new methods represent largely incremental or marginal improvements over previous methods, as was observed in a recent review comparing them [40]. Alternatively, it might suggest that the ∼60% accuracy achieved by these methods represents the theoretical limit to what can be predicted from sequence and limited structural information alone. Further prospective validation of these prediction methods on truly novel antigens is needed to more fully determine our capability to predict B cell epitopes.

Prediction of T cell reactivity revealed several CD4^+^ and CD8^+^ epitopes distributed over the entire *Pf*CelTOS molecule, but *in vivo* functional (ELISpot) analyses indicated that only C-terminal epitopes were immunogenic in mice. Analysis of predicted T cell epitopes revealed one CD8^+^ epitope nested within a CD4^+^ T cell epitope (AA 148–156 within the AA 145–153) specific for BALB/c mice. Interestingly, the region of AA 133–180 contains three CD8^+^ T cell epitopes (H2-K^d^) and three CD4^+^ T cell epitopes (I-E^d^) for BALB/c mice and their functional activity is reflected by the strong T cell responses measured by ELISpot. The same region contains one CD8^+^ T cell epitope (H2-K^b^) and three CD4^+^ T cell epitopes (I-A^b^). T cell responses in ICR mice differed from those in inbred mice by additional, albeit moderate activity at the N-terminal portion of the molecule.

### Conclusion

Current *in silico* computational methods used for predicting T cell and B cell epitopes have varying degrees of accuracy for predicting linear and discontinuous B cell epitopes. In order to improve the accuracy of these predictions it is essential to “train” the algorithms by using the responses achieved in wet lab *in vivo* experimental models. The comparisons conducted in the present study indicate that the available methods are not quite there and that optimized algorithms will be invaluable for the design of next generation, efficacious vaccines.
